# Colostomy for Bowel Diversion in Aggressive Diverticulitis With Colovesical and Colocutaneous Fistulae: A Case Report

**DOI:** 10.7759/cureus.103173

**Published:** 2026-02-07

**Authors:** Arman Haveric, Juan Poggio, Mauricio Wilk

**Affiliations:** 1 Department of Surgery, Lewis Katz School of Medicine at Temple University, Philadelphia, USA

**Keywords:** case report, colocutaneous fistula, diverticulitis, sigmoid colon, transverse loop colectomy

## Abstract

Fistulizing diverticulitis is an uncommon and aggressive manifestation of diverticular disease. Most patients develop a single fistula; multiple synchronous fistulae are less commonly reported. We present a case of a 55-year-old man with a history of anorectal fistula repair who presented with recurrent abdominal pain, urinary symptoms, and weight loss. Imaging revealed complicated sigmoid diverticulitis with multiple fistulae involving the bladder, rectum, and abdominal wall. Initial management included abscess drainage and a diverting transverse loop colostomy, which successfully controlled sepsis and halted fistula output. After clinical optimization, the patient underwent open sigmoid resection, colovesical fistula takedown, and bladder repair, followed by colostomy reversal. At follow-up, he remained asymptomatic with normal bowel function. This case highlights an unusual presentation of complicated diverticulitis and demonstrates the effectiveness of a staged approach using transverse loop colostomy prior to definitive resection.

## Introduction

Diverticulitis is a common condition in older adults, but its fistulizing variant is far less frequent and often presents significant management challenges [1]. Fistulizing diverticulitis occurs in approximately 1-4% of patients with acute diverticulitis, and among those who develop fistulae, the majority present with a single tract [2-4]. Recently, fistulizing diverticulitis has been proposed as a distinct clinical phenotype of diverticular disease, in that it shares histopathologic characteristics with Crohn’s disease, exhibits wide anatomic variability of fistula locations, and predominantly requires surgical management [5]. The distribution of fistula types in fistulizing diverticulitis is predominantly colovesical (approximately 65%) and colovaginal (25%) [2], while colocutaneous and complex multiorgan fistulae comprise a much smaller proportion, previously reported as 4% [6] but more recently reported closer to 10% when grouped with complex fistulae [2]. The simultaneous presence of colorectal, colovesical, and colocutaneous fistulae therefore represents an exceptionally uncommon clinical scenario, with no representation in the literature that we searched.

Here we describe a rare case of sigmoid diverticulitis with acute pelvic abscess formation. Drainage of the abscess was complicated by colocutaneous fistulae, and was successfully managed with a staged surgical approach, utilizing transverse loop colostomy and subsequent definitive resection, at an academic medical center. While to our knowledge, there are no specific systematic reviews or meta-analyses specifically addressing diverticulitis with multiple synchronous fistulae, several recent studies have described one-stage resection with primary anastomosis as the standard of care [7,8]. Our case contributes to the literature by highlighting an infrequently described diversion strategy necessitated by patient-specific anatomy and physiology in the management of Hinchey II diverticulitis [9] with contained abscess formation.

## Case presentation

Our patient is a 55-year-old-male patient with a prior medical history of fistulizing diverticulitis (previously managed with a bio-LIFT [10] for anorectal fistula), hypertension, and asthma. He presented to the emergency department with acute lower abdominal pain. CT imaging in 2021 has previously revealed an acute-on-chronic diverticulitis with multiple fistulas, involving the sigmoid colon, bladder dome, rectum, and left rectus musculature. Symptoms improved with antibiotics, but the patient declined surgical follow-up. In 2023, the patient underwent a diagnostic colonoscopy, with one benign rectal polyp removed, but the procedure was aborted due to significant stenosis of the sigmoid colon. The patient was formally recommended to undergo a CT abdomen and pelvis with contrast for further diagnostic workup, but did not follow up.

Prior to his presentation, the patient had remained intermittently symptomatic, reporting progressive weight loss, irregular bowel function, recurrent lower abdominal pain, dysuria, and pneumaturia, consistent with his known history of fistulizing diverticulitis.

In June 2024, the patient presented to the emergency department with worsening left inguinal pain, which had begun two weeks earlier, after the patient had been weightlifting. On physical examination, the patient was noted to have an extensive and severe inflammatory reaction (edema, erythema, tenderness, and fluctuance) over the left lower quadrant and inguinal region. The patient was afebrile and hemodynamically stable, though he appeared acutely ill and satisfied the Systemic Inflammatory Response Syndrome (SIRS) criteria (due to acute tachycardia and leukocytosis) (Tables 1, 2).

**Table 1:** The patient's vital signs at presentation

	Patient's value	Reference range
Temperature	98.1 °F (36.7 °C)	36.5–37.5 °C (97.7–99.5 °F)
Heart rate	92 BPM	60-100 BPM
Respiratory rate	20 breaths/min	12-20 breaths/min
Blood pressure	123/66 mmHg	<120/<80 mmHg

Laboratory workup additionally revealed anemia, though lactate was within normal limits. Blood cultures were drawn, and showed no growth after five days (Table 2).

**Table 2:** The patient's laboratory values on initial presentation

	Patient's value	Reference range
Leukocyte count	13,600 cells/μL	4,000 - 11,000 cells/μL
Hemoglobin	11.7 g/dL	13.5 - 17.5 g/dL (males)
Lactate	1.7 mmol/L	0.5 - 2.22 mmol/L

**Figure 1:**
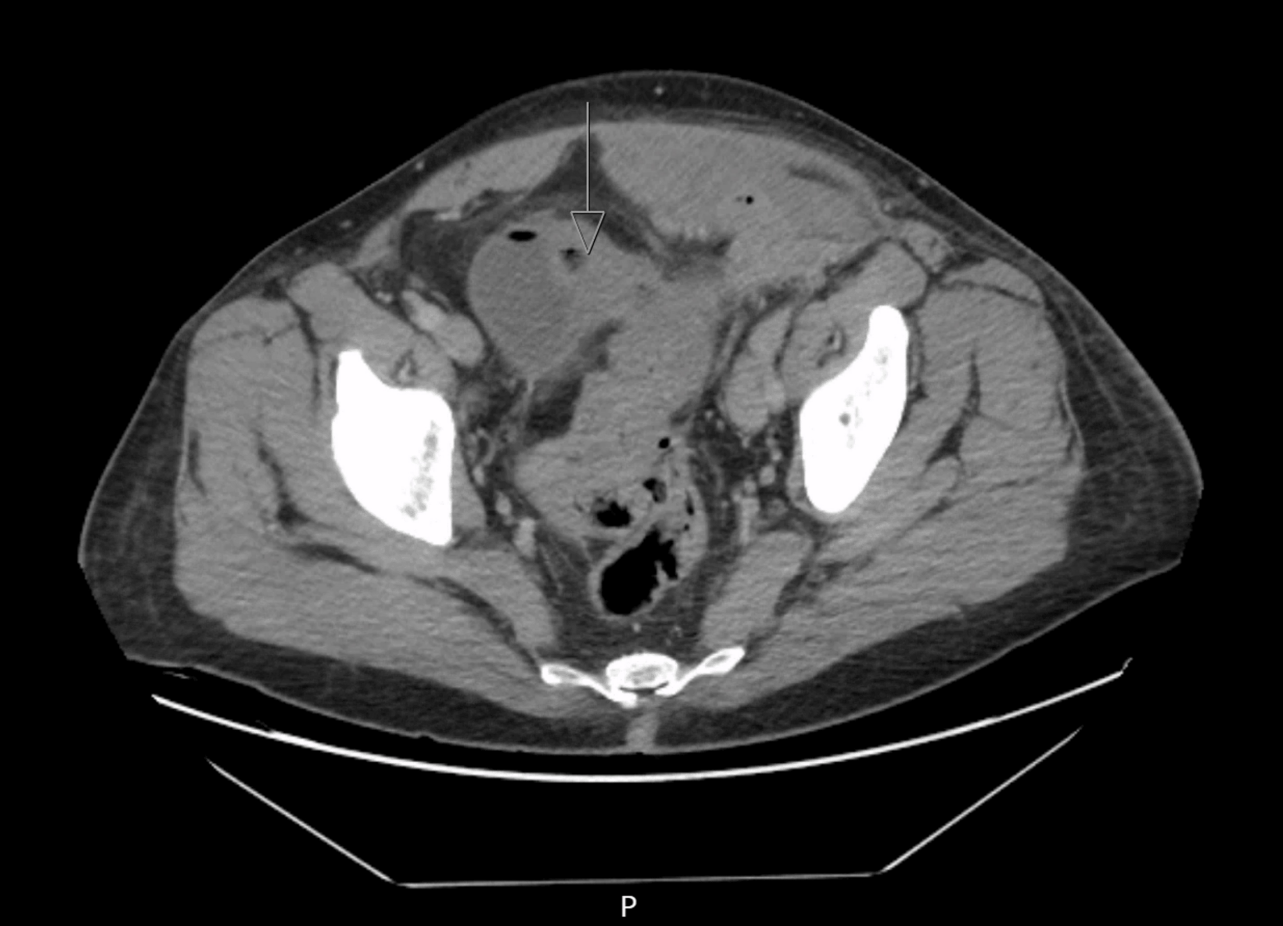
Intraluminal air in urinary bladder, consistent with a colovesical fistula

**Figure 2:**
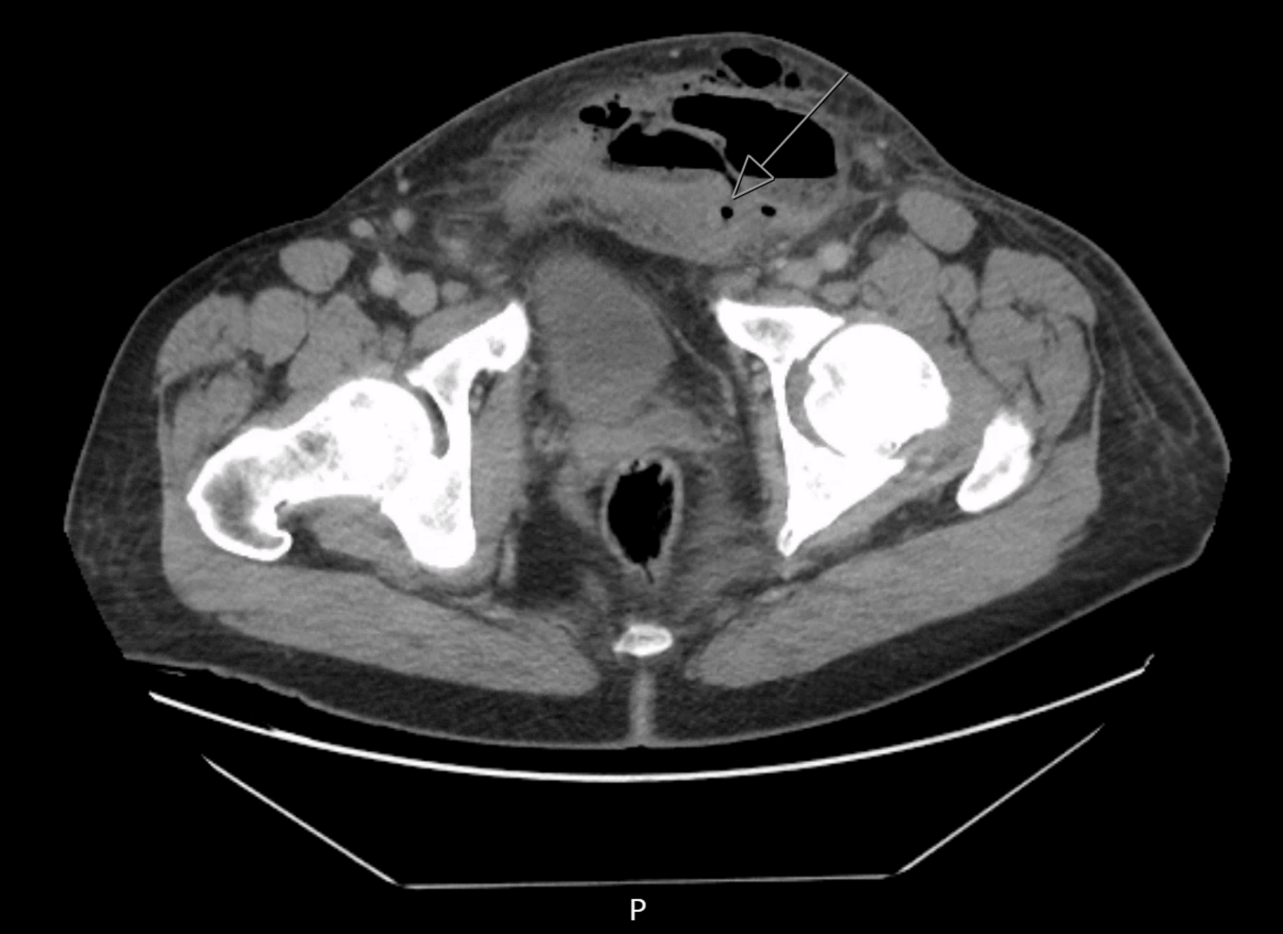
Extensive phlegmonous changes in the abdomen containing air and fluid

CT abdomen/pelvis was suggestive of communicating tracts between the sigmoid colon and left lower abdominal wall with extensive phlegmonous changes containing air and fluid, consistent with a Hinchey II diverticulitis [9] classification (Figures 1, 2). 

The radiologist's review suggested a contained perforation of complicated sigmoid diverticulitis with abscess formation, involving the left lower quadrant abdominal wall and left inguinal region with the possibility of a strangulated hernia, however without proximal colonic obstruction. Additionally, there was evidence of a colovesical fistula. No intraperitoneal free air or fluid was observed.

The patient was adequately resuscitated with IV fluids, received broad spectrum antibiotic therapy with piperacillin-tazobactam, and the same day underwent incision and drainage of the left lower quadrant/inguinal abdominal wall abscess which yielded abundant purulent material, albeit with no evidence of stool or obvious bowel involvement. Considering the operative findings of a contained process, a benign abdominal exam, normal lactate, and hemodynamic stability, further surgical exploration was not pursued. The initial management decision was to control the patient’s acute septic presentation via drainage of the contained abscess.

The following day, the patient was noted to have liquid stool drainage from the incision site, representing the formation of a colocutaneous fistula. The patient was initially conservatively managed with regular wound packing changes, continued IV antibiotics, and clear liquid diet to minimize stool burden. At this stage, his surgical team reasoned that the degree of inflammation and risk for intraperitoneal stool leakage made primary resection and anastomosis unfavorable. Two days later, the patient underwent a diverting transverse loop colostomy with the intention of allowing the inflammatory process in the sigmoid colon to resolve prior to eventual resection. The left inguinal wound site was irrigated and lightly packed with gauze and was stabilized with a wound manager appliance. Output from the colocutaneous fistula stopped the day following surgery as the colostomy became functional. The patient made good postoperative progress and was educated on ostomy and wound care. He was discharged from the hospital five days following the procedure. Wound cultures during his initial incision and drainage speciated *Enterococcus faecalis* without high-level aminoglycoside resistance.

**Figure 3:**
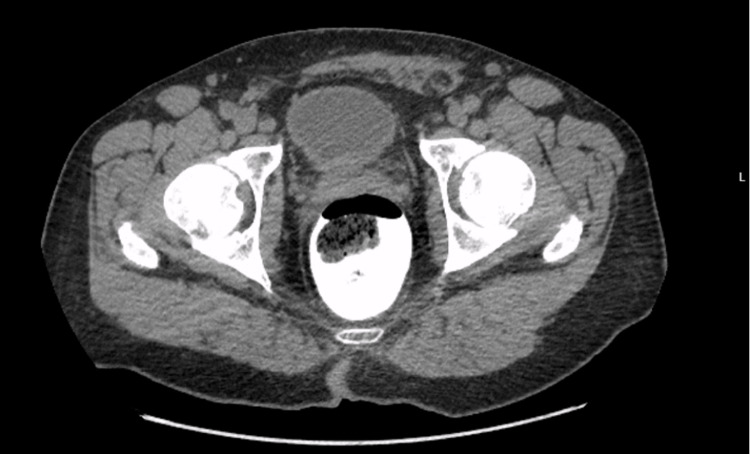
Computed tomography of the abdomen/pelvis with rectal contrast Taken four months after index case, the image demonstrates marked resolution of soft tissue phlegmononous changes.

The patient was noted to have a favorable postoperative progress on outpatient follow-up with resolution of urinary symptomatology, anemia, and malnutrition. CT abdomen/pelvis with rectal contrast, obtained four months after discharge from the hospital, demonstrated significant improvement and almost complete resolution of the acute sigmoid and abdominal wall inflammatory process (Figure 3).

In preparation for sigmoidectomy, the patient underwent colonoscopy, both trans-rectally and through the efferent limb of his loop ostomy site. Colonoscopy revealed severe sigmoid stenosis that could not be traversed. No polyps or masses were appreciated on this examination

Nine months after discharge from the hospital, the patient underwent a challenging sigmoid resection with primary anastomosis. This was accomplished via an open laparotomy, which was favored as a surgical technique due to extensive adhesions from the chronic inflammatory process, and 21 centimeters of sigmoid colon were resected. The descending colon was adequately mobilized and anastomosed to the rectum. The original diverting transverse loop colostomy was preserved in order to protect the anastomosis [11]. The operative procedure included placement of ureteral stents, takedown of the colovesical fistula, and bladder repair. Given the patient’s history of multiple fistulae and a sigmoid stricture, Crohn’s disease was considered in the differential diagnosis. However, histopathologic analysis of the surgical specimen revealed submucosal fibrosis (consistent with history of chronic inflammation) and serosal hemorrhage within a fistula tract, albeit without any further indications of inflammatory bowel disease. Outpatient colonoscopic surveillance was planned following clinical stabilization.

The postoperative progress was favorable. Two months after sigmoid resection, the patient underwent an uncomplicated closure of the transverse loop colostomy. The postoperative progress was unremarkable. The patient has remained asymptomatic and with normal bowel function during the one- and three-month follow-up as an outpatient.

Four months after the colostomy closure, the patient underwent a successful screening biopsy that revealed perianal skin tags and some hemorrhoids but no fistulae. A five-millimeter, semi-pedunculated polyp was found in the proximal rectum which was excised and was found to be benign on pathologic reviews. Colonoscopy was otherwise grossly negative for ongoing inflammation or stricture.

## Discussion

Our patient’s clinical history and operative management contain several unique elements. First, although fistulizing diverticulitis is well-documented in the literature, clinically, majority of patients present with a single fistula, not multiple ones. A retrospective review of 42 patients admitted for the management of fistulizing diverticulitis showed that 14% of included patients experienced multiple fistulae [6]. To our knowledge, our patient’s specific combination of colorectal, colovesical, and colocutaneous fistulae arising from sigmoid diverticulitis has not yet been documented in the literature.

Classically, an end colostomy (Hartmann procedure) or a proximal loop ileostomy are the common interventions used for fecal diversion in patients requiring acute surgical management of diverticulitis [2,12]. The precise technique used is often dependent on the surgeon’s clinical judgment [13]. The use of loop transverse colostomy for the treatment of acute diverticulitis is infrequently described in the literature; however, Gajendran et al. [14] and Nasir et al. [15] have both described its use for temporary fecal diversion in the management of pyelophlebitis secondary to diverticulitis.

Existing systematic review and meta-analysis data only compare primary resection and anastomosis to staged diversion using the Hartmann approach. Two recent studies are of specific interest to our case. First, a 2022 systematic review by Froiio et al. analyzed 1,061 patients with colovesical fistula from 14 studies published between 2014 and 2020 [7]. This review established that one-stage colonic resection with primary anastomosis is preferred over staged diversion via the Hartmann procedure wherever possible. Additionally, a 2023 meta-analysis by Horesh et al. evaluated data from 499 patients in five randomized control trials, demonstrating that primary anastomosis had significantly lower odds of long-term ostomy, complications, and reoperation compared to Hartmann procedures [8].

Importantly, our case contributes by demonstrating that a staged approach using transverse loop colostomy can achieve similar favorable outcomes (patient was ultimately stoma-free) while allowing optimization during the acute inflammatory phase. Although we are not able to generalize from a single case, our patient’s case is illustrative of multiple clinical factors that increased the operative risks of a primary sigmoid resection, and thus justified the staged diversion approach: his peri-diverticular phlegmonous changes, positive sepsis criteria, malnutrition with progressive weight loss, anemia, contained abscess formation, and multiple patent fistulae. Further, his clinical presentation in the absence of colonic obstruction or severe distention allowed proximal diversion, with the goal of optimizing the patient before complex multi-fistula resection.

The clinical superiority of transverse loop colostomy versus ileostomy has not been determined, with many studies comparing the two techniques producing equivocal findings [16]. Indeed, in 2022 the American Society of Colon and Rectal Surgeons (ASCRS) updated its 2015 guidelines to reflect non-superiority of loop ileostomy versus colostomy due to their differing risk-benefit profiles [17]. Thus, patient-specific factors and surgeon judgement must guide the decision for ostomy site in a patient requiring urgent bowel diversion.

## Conclusions

Diverticulitis with multiple synchronous fistulae is an uncommon and challenging presentation for which standardized management strategies are limited. In this case, our patient achieved clinical stabilization through a staged approach involving abscess drainage followed by transverse loop colostomy, and ultimately definitive resection. Although we cannot generalize conclusions from this single patient, this experience suggests that transverse loop colostomy is a reasonable diversion strategy in carefully selected patients without obstruction or severe colonic distention. Delaying definitive sigmoid resection allowed for substantial improvement in the inflammatory burden and overall patient condition prior to complex surgery. This case adds to the limited literature on alternative diversion approaches in severe fistulizing diverticular disease and underscores the importance of individualized surgical planning.
